# Ringworm Clinics

**Published:** 1921-08-20

**Authors:** 


### Ringworm Clinics.

"X-Ray Sister" writes: In view of the active campaign made on ringworm by the London County Council, in conjunction with the larger London hospitals, it seems regrettable that there is no system of post-treatment clinics to supervise the carrying out of instructions given by the hospital authorities to the parents of children affected. Some hospitals do, it is true, employ almoners for this purpose, but they do not carry the same conviction, somehow, as trained nurses. In my experience, when I have had the good fortune to interest a school or district nurse in my little "ringworms," the result has invariably been excellent.

To illustrate the hiatus: A child attends for treatment, receives an epilating dose of *x*-ray, and is taken home by its mother, who bears a little slip of instructions something like the following:

1. To apply the ointment prescribed, renewing it each morning after washing off the old application.
2. To try for loosened hairs in ten or fifteen days' time, and continue till scalp is *clear of hair*.
3. To bring the child in a fortnight for inspection in the department, and in three weeks to the doctor.

Further verbal instructions will be added, laying emphasis on the wearing of washing-caps, the segregation of the child, the importance of instruction 2, etc., etc. The mother goes home fired with enthusiasm—which lasts with an average mother perhaps a week—but which, alas! too often peters out before the epilation stage. A few perfunctory attempts at epilation may be made, probably with fingers instead of tweezers; and I have known an ostrich-like mother shave off the remaining hair when her patience gave out! Other and plentiful pitfalls there are for mothers, such as trying experiments with quack ointments, and the use of irritating soap; others again become so obsessed with the value of the ointment (which is to assist epilation) that they will continue use of it long after the need has passed. This is a superfluity that no doctor can foresee. In short, it is on the safer side when they take the line of least resistance and do nothing at all, although in either case the work of the *x*-ray operator is rendered futile. Could not this handicap to an already thorny path be removed? Would it be feasible to include the *x*-ray treatment of ringworm in a school nurse's curriculum, or, if the little victims are under temporary banishment, could not the district nurse supervise the mothers by arrangement with the L.C.C.? Undoubtedly they would have to be indemnified if such a scheme added to their burdens; but the influence of the trained nurse is so powerful with mothers of the poorer class that one feels their co-operation is worth gaining.

